# Pediatric sepsis inflammatory blood biomarkers that correlate with clinical variables and severity of illness scores

**DOI:** 10.1186/s12950-024-00379-w

**Published:** 2024-03-07

**Authors:** Sean Leonard, Hailey Guertin, Natalya Odoardi, Michael R. Miller, Maitray A. Patel, Mark Daley, Gediminas Cepinskas, Douglas D. Fraser

**Affiliations:** 1 Pediatrics, Western University, London, ON Canada; 2 Emergency Medicine, Lakeridge Health, Ajax/Oshawa, ON Canada; 3 Epidemiology and Biostatistics, Western University, London, ON Canada; 4 Computer Science, Western University, London, ON Canada; 5 Medical Biophysics, Western University, London, ON Canada; 6 Lawson Health Research Institute, London, ON Canada; 7 Clinical Neurological Sciences, Western University, London, ON Canada; 8 Physiology & Pharmacology, Western University, London, ON Canada; 9 Room C2-C82, London Health Sciences Centre, 800 Commissioners Road East, London, ON N6A 5W9 Canada

**Keywords:** Sepsis, Severity, Biomarkers, PICU, Pediatrics, Plasma proteins

## Abstract

**Background:**

Sepsis is a dysregulated systemic inflammatory response triggered by infection, resulting in organ dysfunction. A major challenge in clinical pediatrics is to identify sepsis early and then quickly intervene to reduce morbidity and mortality. As blood biomarkers hold promise as early sepsis diagnostic tools, we aimed to measure a large number of blood inflammatory biomarkers from pediatric sepsis patients to determine their predictive ability, as well as their correlations with clinical variables and illness severity scores.

**Methods:**

Pediatric patients that met sepsis criteria were enrolled, and clinical data and blood samples were collected. Fifty-eight inflammatory plasma biomarker concentrations were determined using immunoassays. The data were analyzed with both conventional statistics and machine learning.

**Results:**

Twenty sepsis patients were enrolled (median age 13 years), with infectious pathogens identified in 75%. Vasopressors were administered to 85% of patients, while 55% received invasive ventilation and 20% were ventilated non-invasively. A total of 24 inflammatory biomarkers were significantly different between sepsis patients and age/sex-matched healthy controls. Nine biomarkers (IL-6, IL-8, MCP-1, M-CSF, IL-1RA, hyaluronan, HSP70, MMP3, and MMP10) yielded AUC parameters > 0.9 (95% CIs: 0.837-1.000; *p* < 0.001). Boruta feature reduction yielded 6 critical biomarkers with their relative importance: IL-8 (12.2%), MCP-1 (11.6%), HSP70 (11.6%), hyaluronan (11.5%), M-CSF (11.5%), and IL-6 (11.5%); combinations of 2 biomarkers yielded AUC values of 1.00 (95% CI: 1.00–1.00; *p* < 0.001). Specific biomarkers strongly correlated with illness severity scoring, as well as other clinical variables. IL-3 specifically distinguished bacterial versus viral infection (*p* < 0.005).

**Conclusions:**

Specific inflammatory biomarkers were identified as markers of pediatric sepsis and strongly correlated to both clinical variables and sepsis severity.

**Supplementary Information:**

The online version contains supplementary material available at 10.1186/s12950-024-00379-w.

## Introduction

Sepsis is a dysregulated systemic inflammatory response triggered by an infection that results in biochemical, physiological, and pathological abnormalities throughout the body, including organ dysfunction [1]. One of the challenges in clinical pediatrics is identifying sepsis, particularly in infants and young children [2]. The ability to efficiently recognize and stratify the severity of sepsis is essential, as it remains a leading cause of morbidity and mortality in the pediatric population [3].

At the time of initial presentation, it can be challenging to identify which children will go on to develop sepsis based on their early clinical features. Indeed, approximately 12% of well-appearing febrile infants have potentially life-threatening serious bacterial infections [4]. Not only can sepsis have negative and devastating consequences for patients, but it remains a significant burden to the healthcare system. In fact, it has been estimated that sepsis treatment in the United States healthcare system costs more than 20 billion dollars annually [5].

Biomarkers are measurable compounds in the body that reflect physiologic states or pathologic processes. A diagnostic test for sepsis that uses novel biomarkers in combination with or without clinical signs would contribute to prompt and appropriate targeting of monitoring and treatment for children at the highest risk [6]. To be effective, compounds measured as biomarkers should be sensitive, accurate, and reproducible [7]. To date, only CRP [8] and procalcitonin [9] are helpful markers of infection and inflammation. Inflammatory biomarkers, such as IL-10, MCP-1, TNF-α, and others, have been implicated in the early development of sepsis but have not been well investigated [7]. Rather than endeavoring to identify one biomarker that will prove reliable in all clinical scenarios to diagnose sepsis, research suggests that the use of several biomarkers in tandem may be more sensitive and specific than a single biomarker [10].

In this study, we performed immunoassays to measure 58 blood inflammatory biomarkers from pediatric sepsis patients and age- and sex-matched healthy control subjects, followed by data analyses with both conventional statistics and machine learning. Our specific objectives were: (1) to measure the plasma levels of biomarkers known to be implicated in sepsis in critically ill children admitted to a pediatric intensive care unit (PICU); (2) to compare those same biomarkers with healthy age- and sex-matched control subjects; (3) to determine how biomarker levels vary over two days in the PICU; and (4) to correlate biomarker concentrations with clinically relevant variables.

## Methods

### Study participants and blood sampling

Consent for research was obtained from the legal guardians of pediatric patients. Patients who met pediatric sepsis criteria were enrolled. Blood was drawn only when clinically required, using citrate as an anticoagulant. Patient characteristics included age, sex, comorbidities, pathogen, source of infection, chest x-ray, mechanical ventilation, hemodynamic support, number of PICU days, and total number of hospital days. Illness severity scores were calculated, including the Pediatric Risk of Mortality (PRISM III) score, the Pediatric Index of Mortality 2 (PIM 2) score, the daily Pediatric Logistic Organ Dysfunction 2 (PELOD-2) score, and the Glasgow Coma scale (GCS). For comparison with sepsis patients, age- and sex-matched healthy control subjects without disease or acute illness were identified from the Translational Research Centre, London, Ontario (www.translationalresearch.ca) [11, 12]. To obtain the plasma, the blood was centrifuged at 1500 × g for 15 min at 4^o^C. Samples were then frozen and stored at − 80 °C before thawing on ice for experimental use.

### Plasma immunoassays

Analyte concentrations of 58 inflammatory analytes were determined using either multiplexed biomarker immunoassay kits according to manufacturers’ instructions (MilliporeSigma, 400 Summit Drive, Burlington, MA) or enzyme-linked immunosorbent assay (ELISA). For the former, plasma inflammatory analytes were measured using a Bio-PlexTM 200 Suspension Array system (Bio-Rad Laboratories, Hercules, CA), which used Luminex xMAPTM fluorescent bead-based technology (Luminex Corp., Austin, TX). Bioanalyte concentrations were calculated from standard curves using five-parameter logistic regression in Bio Plex Manager 6.1 software. For the latter, the biomarkers measured with ELISA include: syndecan-1 (1:2 dilution; ab46506, Abcam, Cambridge, UK); heparin sulfate (1:10 dilution; HU8718, Biotang Inc., Lexington, MA); chondroitin sulfate (1:2 or 1:50 dilution; HU8720, Biotang Inc.); and hyaluronan (1:8 dilution; DHAYL0, R&D Systems, Minneapolis, MN). Plasma samples were assessed in duplicate, and where sample optical densities (ODs) were above the range of the plate reader, concentrations were estimated by substituting the highest OD value of the plate reader. Plasma biomarker data are presented as pg/mL.

### Conventional statistics

Medians with interquartile ranges (IQRs) and frequency (%) were used to report PICU patient baseline characteristics for continuous and categorical variables, respectively. Group differences for continuous and categorical variables were examined with Mann-Whitney U tests (or Kruskal Wallis tests, as appropriate) and chi-square tests, respectively. Differences between sepsis patients on PICU Day-1 and Day-2 were examined with Wilcoxon signed-rank tests. Receiver operating characteristic (ROC) curves were estimated for individual analytes to predict sepsis status in comparison to healthy control subjects [13]. Area-under-the-curve (AUC) was calculated as an aggregate measure of analyte performance across all possible classification thresholds, with AUC > 0.7 considered acceptable [14]. Analyte combinations were calculated through logistic regression models with sepsis status as the outcome and representative analytes as the included predictors; the predicted values from the regression models were then saved for use in ROC curve analyses. All analyses were conducted using SPSS version 28 (IBM Corp., Armonk, NY). Heat maps depicting Pearson correlation values between analytes and clinical or biochemical parameters were created in R (http://www.r-project.org) using the ggplot2 version 3.3.3 package. Statistical significance was set at a *p*-value < 0.01.

### Machine learning

For machine learning, a Random Forest classifier based on decision trees was used to classify the sepsis cohort in comparison to the healthy control subjects by their biomarker concentrations. The Boruta feature reduction algorithm was used to identify the most important biomarkers for classifying sepsis [15]. The Boruta algorithm is based on Random Forest classifiers and compares the original biomarker dataset to randomly rearranged versions to determine if a biomarker is better at classifying than chance. The biomarker data were then visualized with a nonlinear dimensionality reduction on the reduced dataset using the t-distributed stochastic nearest neighbor embedding (t-SNE) algorithm. t-SNE assumes that the ‘optimal’ representation of the data lies on a manifold with complex geometry but a low dimension, embedded in the full-dimensional space of the raw data [16].

## Results

Two age- and sex-matched groups of 20 pediatric patients were enrolled in the study, including a group of pediatric patients with sepsis (median age = 13 years) and a group of healthy control subjects (Table 1). Plasma samples were collected from pediatric sepsis patients on days one and two of admission to the PICU. Only 13 sepsis patients remained in the PICU on Day-2. Baseline characteristics of these patients, including PRISMIII score, PIM2 score, daily PELOD-2 score, GCS, comorbidities, and infection data, are found in Table 1. Vasopressors were administered to 17 of the 20 patients (85%); 11 patients (55%) received invasive ventilation by intubation or tracheotomy; and 4 patients (20%) were ventilated non-invasively by BiPAP, CPAP, or high-flow nasal cannula.

**Table 1:** Baseline characteristics of 20 pediatric sepsis patients

Age, y		13 (8.5, 15)		
Sex, female		6 (30%)		
Comorbidities				
Neurology		10 (50%)		
Oncology		3 (15%)		
Respiratory		2 (10%)		
Genitourinary		2 (10%)		
Diabetology		2 (10%)		
Cardiology		1 (5%)		
Gastrointestinal		1 (5%)		
None		6 (30%)		
Sepsis, proven		15 (75%)		
Sepsis, suspected		5 (25%)		
Pathogens				
Gram-negative bacteria		7 (35%)		
Gram-positive bacteria		4 (20%)		
Viral		3 (15%)		
Fungal		2 (10%)		
Unknown		6 (30%)		
CXR abnormalities, present		14 (70%)		
Source of Pathogen				
Respiratory		11 (55%)		
Cardiovascular		4 (20%)		
Gastrointestinal		2 (10%)		
Genitourinary		1 (5%)		
Wound		1 (5%)		
Unknown		1 (5%)		
PRISM III score		5 (3, 13.25)		
PIM-2 mortality risk		-3.96 (-4.44, -3.17)		
PELOD-2 score, initial		11 (4, 18)		
PELOD-2 score, highest		12 (11, 21)		
GCS on admission		13 (6, 15)		
Invasive Ventilation		11 (55%)		
Non-Invasive Ventilation		4 (20%)		
Inotrope/vasopressor administered		17 (85%)		
PICU days		4.5 (1.25, 10)		
Hospital days		15.5 (9, 35.75)		

Data presented as median (IQR) or n (%)

A total of 58 plasma protein biomarkers were measured using immunoassays, with 24 being statistically significantly different between the two cohorts (Table 2). Medians and IQRs for these biomarkers are shown in descending order of significance and are represented in pg/mL. Most biomarkers had significant elevations in concentration on PICU Day-1 when compared to the healthy controls (*n* = 21/24; *p* < 0.01), with only 3 biomarkers decreased in sepsis patients (RANTES, MMP9, and MMP2). A total of 34 of the measured plasma proteins did not reach statistical significance (Supplementary Table 1).

**Table 2:** Statistically significant plasma biomarker measurements

Biomarker	Healthy Controls (*n* = 20)	PICU Day-1 Sepsis (*n* = 20)	PICU Day-2 Sepsis (*n* = 13)	*P*-value
G-CSF	0 (0, 56.97)	498.26 (219.98, 5422.09)	426.49 (104.57, 681.23)	< 0.001
IL-1RA	8.32 (3.71, 17.67)	231.23 (126.02, 1075.80)	75.22 (29.60, 224.32)	< 0.001
IL-6	0.67 (0.32, 1.68)	288.22 (59.45, 3472.15)	96.09 (24.35, 311.18)	< 0.001
IL-8	1.70 (1.10, 3.58)	12.79 (6.30, 249.72)	8.70 (5.98, 180.31)	< 0.001
IL-10	3.16 (0, 26.16)	132.62 (30.15, 868.09)	74.32 (23.20, 309.52)	< 0.001
IL-18	39.95 (28.15, 58.54)	87.32 (59.95, 116.55)	86.58 (76.50, 115.00)	< 0.001
MCP-1	224.6 (187.1, 281.8)	1273.3 (724.8, 3637.4)	842.7 (617.1, 4619.6)	< 0.001
M-CSF	14.25 (0.51, 42.50)	240.44 (141.53, 474.90)	209.70 (119.20, 539.35)	< 0.001
MIP-1β	25.98 (19.61, 35.02)	56.85 (38.73, 131.75)	71.72 (38.33, 112.04)	< 0.001
TNFα	22.20 (10.11, 53.54)	119.82 (41.25, 384.94)	73.57 (36.37, 339.42)	< 0.001
RANTES	10,590 (6150, 15,275)	4811 (1939, 6417)	4121 (1356, 4597)	< 0.001
MMP3	7414 (3751, 10,487)	28,604 (21,388, 45,900)	28,600 (19,827, 40,895)	< 0.001
MMP1	413.7 (367.6, 471.7)	1374.5 (761.0, 3800.6)	1099.4 (536.7, 1425.0)	< 0.001
MMP7	3892 (3279, 4360)	7563 (4528, 11,636)	7771 (6136, 13,026)	< 0.001
MMP10	444.2 (312.2, 616.1)	2039.1 (971.6, 4170.0)	2033.7 (1297.2, 4587.0)	< 0.001
Elastase 2	3.01 (1.60, 4.19)	21.23 (13.72, 58.22)	19.79 (8.71, 33.05)	< 0.001
HSP70	21,451 (19,626, 32,424)	124,090 (64,321, 179,781)	144,242 (69,428, 239,501)	< 0.001
MMP8	372.5 (217.7, 513.6)	5089.1 (1129.6, 19915.9)	6009.0 (1623.7, 15444.8)	< 0.001
Hyaluronan	20.10 (14.85, 46.24)	137.98 (85.20, 362.31)	106.24 (55.31, 538.60)	< 0.001
NGAL	60.86 (45.58, 95.74)	223.05 (132.35, 1651.90)	294.48 (134.41, 2063.79)	< 0.001
Resistin	9.06 (6.41, 11.71)	61.39 (19.75, 119.30)	30.57 (17.70, 62.13)	< 0.001
MIG	1295 (1013, 2772)	3665 (1959, 13,735)	2776 (1947, 5235)	0.004
MMP9	16,018 (13,471, 18,928)	10,235 (6335, 17,943)	8586 (5882, 15,967)	0.008
MMP2	115,962 (99,215, 133,619)	98,954 (82,197, 106,700)	90,882 (74,505, 109,475)	0.009

Data presented in pg/mL as median (IQR)

The statistically significant biomarkers measured on PICU Day-1 were first assessed individually to identify their utility as standalone markers of sepsis. ROC curve analyses were performed on the statistically significant biomarkers with AUCs shown in Table 3. AUC values > 0.8 are considered “good”, while values > 0.9 are considered “outstanding” [17]. Nine biomarkers (IL-6, HSP70, MMP3, MCP-1, Hyaluronan, M-CSF, IL-1RA, MMP10, and IL-8) yielded AUC parameters > 0.9 with 95% CIs ranging from 0.84 to 1.00. The *p*-values for these biomarkers were all < 0.001.

**Table 3:** Receiver operating curve analyses for predicting PICU day-1 sepsis

Biomarker	AUC	95% CI	*P*-value
IL-6	0.995	0.982–1.000	< 0.001
HSP70	0.975	0.937– 1.000	< 0.001
MMP3	0.968	0.923–1.000	< 0.001
MCP-1	0.967	0.904–1.000	< 0.001
Hyaluronan	0.965	0.913–1.000	< 0.001
M-CSF	0.943	0.853–1.000	< 0.001
IL-1RA	0.939	0.870–1.000	< 0.001
MMP10	0.927	0.851–1.000	< 0.001
IL-8	0.923	0.837–1.000	< 0.001
G-CSF	0.882	0.775–0.990	< 0.001
MIP-1β	0.880	0.765–0.995	< 0.001
MMP8	0.879	0.757–1.000	< 0.001
MMP1	0.874	0.751–0.996	< 0.001
IL-10	0.868	0.757–0.978	< 0.001
Elastase 2	0.867	0.733–1.000	< 0.001
IL-18	0.855	0.736–0.974	< 0.001
TNFα	0.852	0.729–0.976	< 0.001
NGAL	0.845	0.713–0.977	< 0.001
Resistin	0.842	0.686–0.999	< 0.001
RANTES	0.833	0.695–0.970	< 0.001
MMP7	0.825	0.675–0.975	< 0.001
MIG	0.775	0.623–0.927	0.003
MMP9	0.747	0.572–0.923	0.007
MMP2	0.735	0.572–0.898	0.011

AUC, area-under-the-curve; CI, confidence interval

**Fig. 1:**
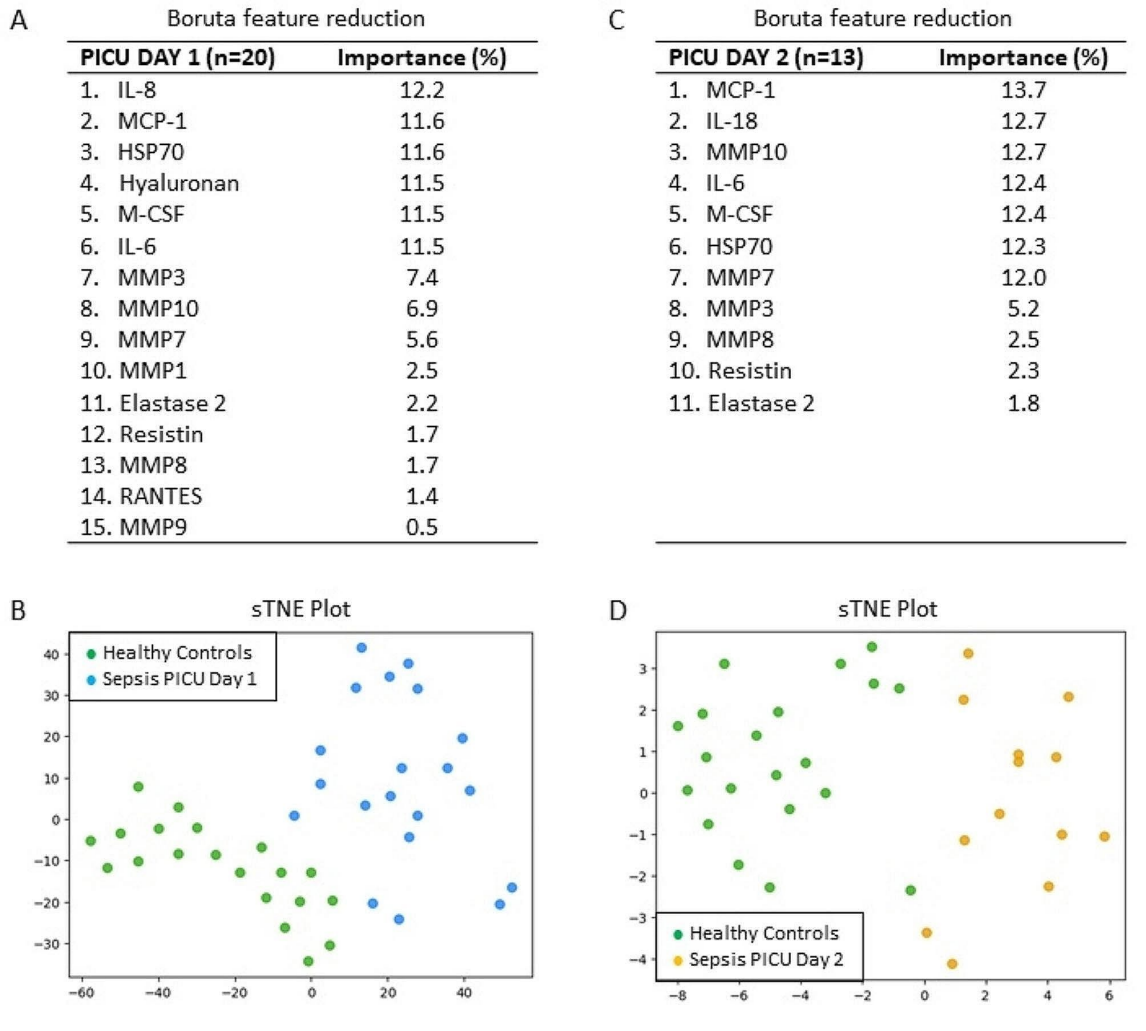
Importance of plasma biomarkers for identifying sepsis on PICUDays 1 and 2. **(A)** Boruta feature reduction reveals the importance of 15 plasma biomarkers for distinguishing sepsis on PICU Day-1. **(B)** Subjects plotted in two dimensions, following t-SNE dimensionality reduction of all 15 significant biomarkers, show distinct separation and clustering of sepsis patients on PICU Day-1 versus age- and sex-matched healthy control subjects. **(C)** Boruta feature reduction reveals the importance of 11 plasma biomarkers for distinguishing sepsis on PICU Day-2. **(D)** Subjects plotted in two dimensions, following t-SNE dimensionality reduction of all 11 significant biomarkers, show distinct separation and clustering of sepsis patients on PICU Day-2 versus age- and sex-matched healthy control subjects.

Machine learning was employed to review the statistically significant biomarkers (as measured on PICU Day-1 and Day-2). The Boruta feature reduction of the leading analytes reduced the number of statistically significant biomarkers to those that were most important (see Fig. 1). On PICU Day-1, of the biomarkers outlined in Table 3, six yielded a biomarker importance in a combined model of > 10%. In descending order, these were IL-8 (12.2%), MCP-1 (11.6%), HSP70 (11.6%), hyaluronan (11.5%), M-CSF (11.5%), and IL-6 (11.5%). On PICU Day-2, the markers shifted, with MCP-1 showing the highest importance (13.7%), followed by IL-18 (12.7%), MMP10 (12.7%), IL-6 (12.4%), M-CSF (12.4%), HSP70 (12.3%), and MMP7 (12.0%), all yielding importance > 10%. Using the combinations of biomarkers in Figs. 1A and C, tSNE plots were constructed (Figs. 1B and D). For PICU Day-1 and Day-2, the tSNE plots showed a clear distinction between the patients with sepsis and the healthy control subjects.

The aforementioned top biomarkers were used in tandem to identify those that could yield AUC parameters even greater than those outlined in Table 3. For combinations of biomarkers on PICU Day-1, the ROC curve analyses are listed in Table 4. Five combinations yielded AUC values of 1.00 (95% CI: 1.00–1.00, *p* < 0.001) and included IL-8 with IL-6, MCP-1 with HSP70, MCP-1 with IL-6, HSP70 with Hyaluronan, and Hyaluronan with IL-6.

**Table 4:** ROC analyses of biomarker combinations on PICU day-1 sepsis

Biomarker Combinations	AUC	95% CIs	*P*-value
IL-8, IL-6	1.000	1.000–1.000	< 0.001
MCP-1, HSP70	1.000	1.000–1.000	< 0.001
MCP-1, IL-6	1.000	1.000–1.000	< 0.001
HSP70, Hyaluronan	1.000	1.000–1.000	< 0.001
Hyaluronan, IL-6	1.000	1.000–1.000	< 0.001
HSP70, IL-6	0.998	0.989–1.000	< 0.001
M-CSF, IL-6	0.995	0.982–1.000	< 0.001
IL-8, HSP70	0.978	0.942–1.000	< 0.001
HSP70, M-CSF	0.975	0.937–1.000	< 0.001
IL-8, Hyaluronan	0.965	0.913–1.000	< 0.001
MCP-1, Hyaluronan	0.965	0.897–1.000	< 0.001
Hyaluronan, M-CSF	0.965	0.913–1.000	< 0.001
IL-8, MCP-1	0.962	0.890–1.000	< 0.001
MCP-1, M-CSF	0.962	0.890–1.000	< 0.001
IL-8, M-CSF	0.953	0.877–1.000	< 0.001

AUC, area-under-the-curve; CI, confidence interval

**Fig. 2:**
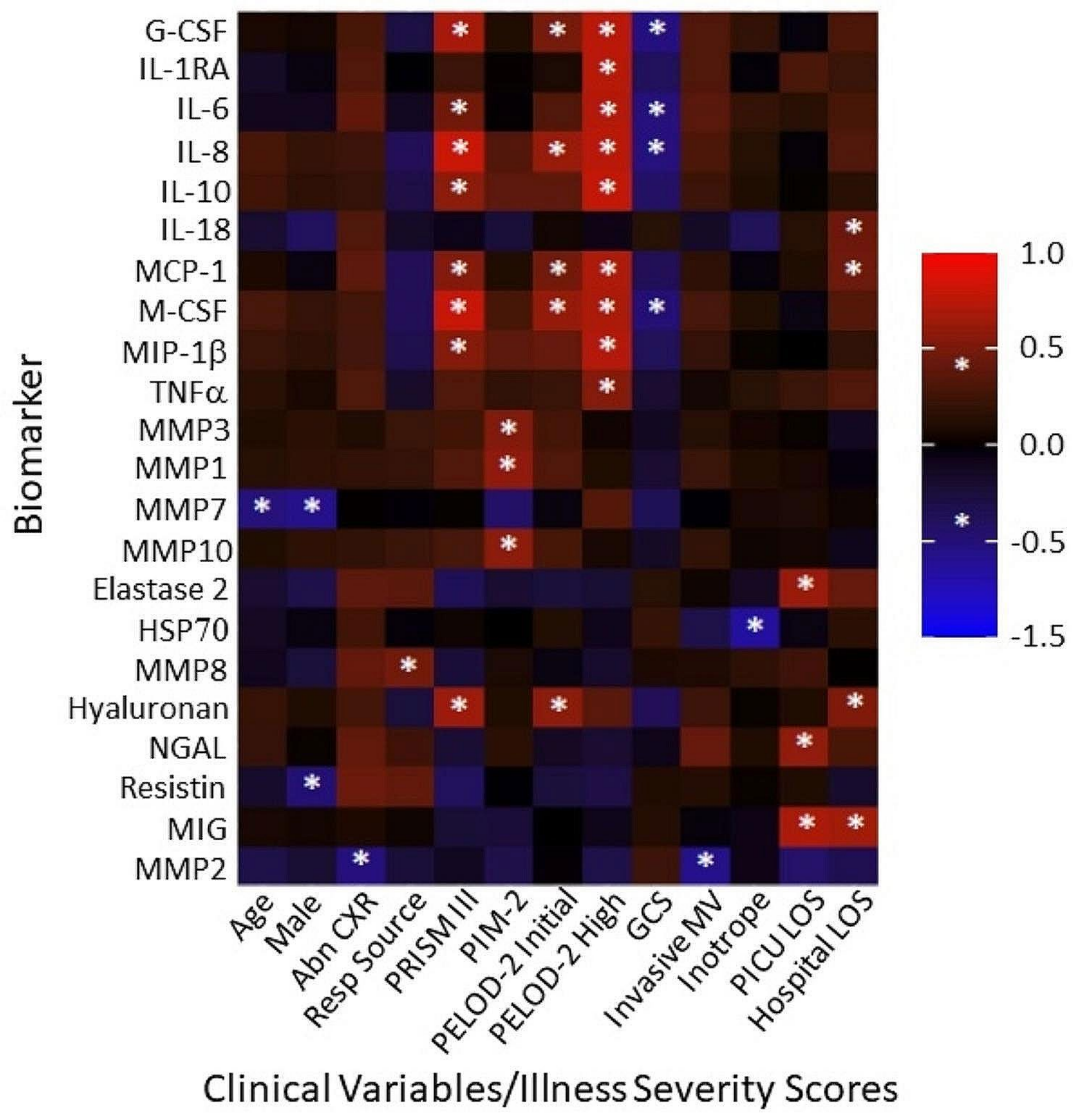
Significant correlations between PICU Day-1 plasma biomarkers and clinical variables/illness severity scores. Biomarker concentrations on the y-axis correlated with clinical variables and illness severity scores. *Abn CXR* reflects any infiltrate identified in either lung field on chest x-ray. *Resp Source* refers to any infectious pathogen identified in the nasopharynx or by respiratory culture. *Invasive MV* refers to any patient requiring mechanical ventilation via either an endotracheal tube or tracheostomy. *LOS* refers to the number of days in either the PICU or hospital. * *p* < 0.05

Statistically significant sepsis biomarkers on PICU Day-1 were correlated with clinically significant variables and severity scores (Fig. 2). Only biomarkers with at least one clinically significant correlation are shown (a total of 22 biomarkers). Age and male sex were negatively correlated with MMP7. Multiple biomarkers demonstrated strong positive correlations with PRISM III and PELOD-2 scores, including G-CSF, IL-1RA, IL-6, IL-8, IL-10, MCP-1, M-CSF, MIP-1β, and hyaluronan. In contrast, PIM 2 scores shared positive correlations selectively with MMPs, including MMP3, MMP1, and MMP10. GCS was negatively correlated with G-CSF, IL-6, IL-8, and MCSF. MMP2 negatively correlated, and MMP8 positively correlated, with respiratory-associated variables. HSP70 was negatively correlated with inotrope requirements. PICU stay was positively correlated with elastase 2, NGAL, and MIG, whereas hospital stay was correlated with IL-18, MCP-1, hyaluronan, and MIG.

**Fig. 3:**
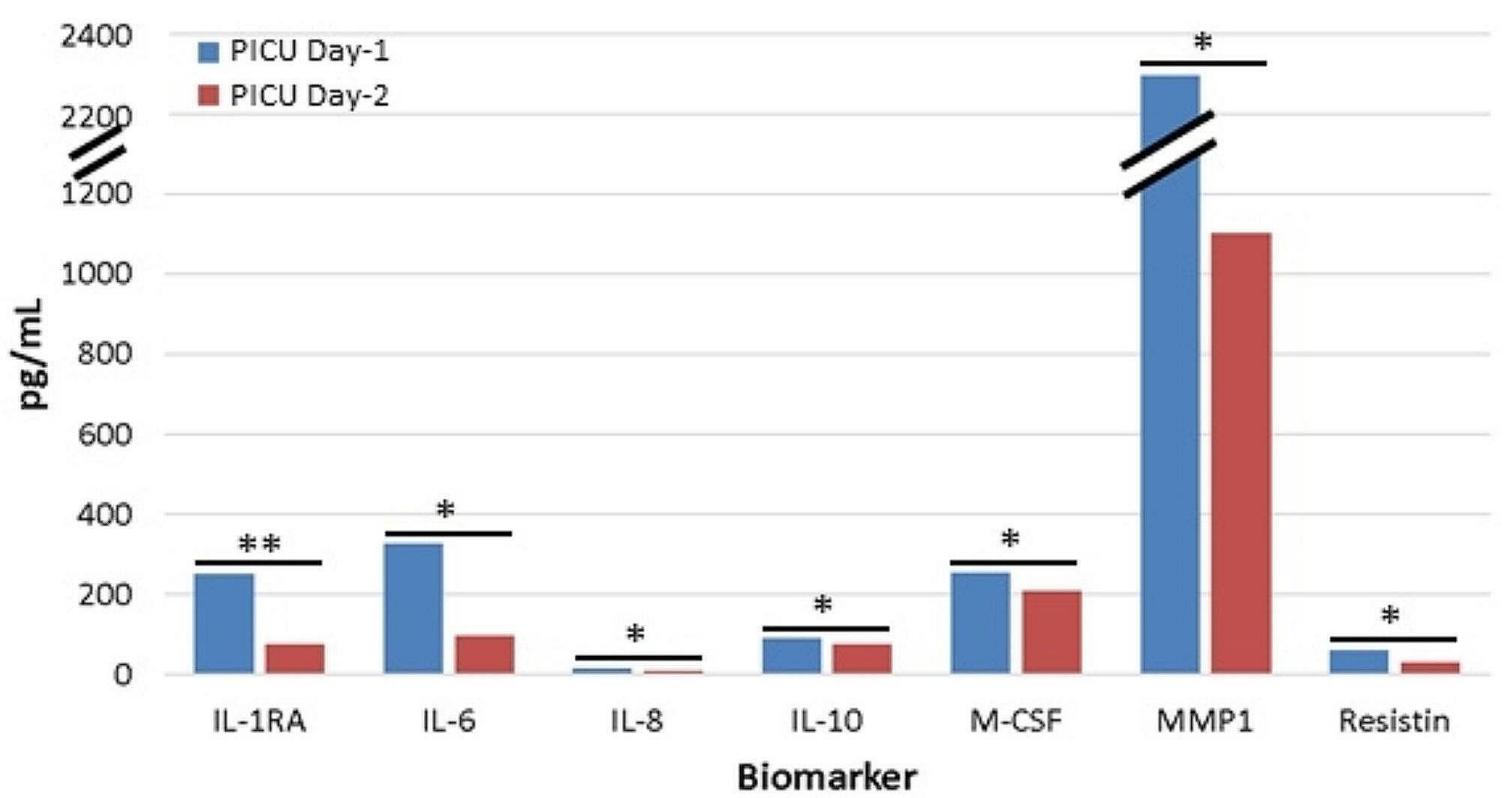
Plasma biomarkers showed significant recovery on PICU Day-2 from their initial peak elevations. Seven plasma biomarkers that were significantly elevated on PICU Day-1 from control levels were significantly decreased by PICU Day-2. Only 13 patients were included for paired analyses and to account for PICU Day-2 discharges where samples were not obtained. * *p* < 0.05, ** *p* < 0.01

A total of seven plasma biomarkers significantly changed over the first 48 h of PICU admission, with all decreasing on PICU Day-2 (Fig. 3). IL-1RA was the most recovered from its peak elevation on PICU Day-1 (*p* < 0.01), while IL-6, IL-8, Il-10, M-CSF, MMP1, and Resistin had moderately recovered from their peaks (*p* < 0.05).

We investigated whether any biomarkers could distinguish between pathogens. Interestingly, IL-3 was not statistically significant between all sepsis patients and healthy control subjects (*p* = 0.428; Supplementary Table 1). However, when plasma IL-3 concentrations from sepsis patients were compared between patients with bacterial (median = 0.00 pg/mL; *n* = 11) versus viral (median = 1.30 pg/mL; *n* = 3) infections, statistical significance was observed (*p* < 0.005).

## Discussion

In this study, we evaluated the plasma protein concentrations of 58 biomarkers in 20 pediatric sepsis patients admitted to the PICU and compared their biomarker levels to those of 20 age- and sex-matched healthy control subjects. Multiple plasma biomarkers were significantly increased in sepsis patients, with nine biomarkers yielding AUC values of > 0.9 on their respective ROC curves. Moreover, combinations of these biomarkers yielded AUC values of 1.00, with five combinations reaching this parameter: IL-8 & IL-6; MCP-1 & HSP70; MCP-1 & IL-6; HSP70 & Hyaluronan; and Hyaluronan & IL-6. Lastly, many biomarkers were correlated with clinical variables and severity of illness scores, suggesting prognostic utility.

Our patients were similar to other pediatric sepsis studies in demographics, illness severity, and clinical interventions [18, 19]. The inciting pathogens were variable, including bacterial, viral, and fungal, with a primary respiratory source. Systemic inflammation and immune dysfunction are characteristic features of pediatric sepsis [20]. A pro-inflammatory stage occurs early, characterized by the recruitment of leukocytes to infected tissues [21]. Once activated, the recruited cells secrete additional pro-inflammatory cytokines [22], chemokines [23], and reactive oxygen species [24]. Local accumulation of these proinflammatory mediators likely instigates microvascular endothelial injury and glycocalyx shedding (e.g., hyaluronan) [25, 26].

Conventional statistical techniques identified 24 plasma biomarkers that were significantly different in sepsis patients from controls. The majority of elevated biomarkers were either pro-inflammatory cytokines [27, 28] or MMPs [29], zinc-containing endopeptidases capable of degrading extracellular matrix proteins and processing bioactive molecules [30]. While 21 biomarkers increased in sepsis, 3 biomarkers decreased from control levels (RANTES, MMP9, and MMP2). Decreased RANTES (or CCL5), a chemokine that regulates CD8 T-cell responses during viral infection, was also depressed from baseline levels in both neonatal and pediatric sepsis [28, 31]. The expression of several plasma MMPs is altered by sepsis in adults, with elevated MMP9 and depressed MMP2 [32]. The depressed MMP9 measured in pediatric sepsis [33] may represent antibody specificity to latent versus active forms or reflect age-dependent differential sepsis responses.

Using machine learning, six biomarkers (MCP-1, M-CSF, IL-6, MMP3, MMP10, and MMP7) were identified that shared high importance between both groups (PICU Day-1 and PICU Day-2) and may be useful for future studies evaluating biomarkers in pediatric sepsis patients who present later in their course of illness than those captured within this study.

The five biomarker combinations that yielded AUC values of 1.00, are individually known to be associated with sepsis and inflammation. IL-6 is an early pro-inflammatory cytokine that induces the synthesis of acute-phase proteins, stimulates antibody production, affects T-cell development, and promotes the differentiation of non-immune cells [34]. IL-8 and MCP-1 are chemokines that recruit leukocytes [35, 36]. Hyaluronan is a constituent of the microvascular glycocalyx [26] and, in response to inflammatory cytokines, is catalyzed into fragments that promote further inflammation [37]. Unlike proteins involved in inflammatory cascades, HSP70 is released by active secretion or during lysis by necrotic cell death, and its levels are often variable in oxidant states. In addition, HSP70 levels correlate with sepsis mortality [38].

We identified multiple sepsis biomarkers that correlated with clinically significant variables. MMP7, an enzyme that degrades the extracellular matrix [32], was negatively correlated with age and male sex, making this biomarker more likely to be present in pediatric sepsis patients who are young and female. MMP8, a neutrophil collagenase, is positively correlated with a respiratory source of infection [39]. The presence of MMP2 is negatively correlated with having an abnormal finding on a chest x-ray, as well as the need for invasive mechanical ventilation [40]. These findings suggest that patients with higher levels of this biomarker are less likely to have an abnormal chest x-ray and less likely to require invasive mechanical ventilation while hospitalized. Additionally, the presence of HSP70 was negatively correlated with the need for inotropic medication, suggesting that patients with increased HSP70 will have overall greater hemodynamic stability throughout their illness course [41].

Multiple biomarkers infer utility in predicting illness severity when matched to established illness severity scores. The biomarkers with the strongest illness severity correlations were G-CSF, M-CSF, and IL-8. These three biomarkers were each positively correlated with PRISM III scores, initial PELOD-2 scores, and the highest measured PELOD-2 scores. In keeping with their role as sepsis severity biomarkers, each of these biomarkers negatively correlated with GCS on admission. As for other markers of sepsis severity, MMP1, MMP3 [42], and MMP10 were all positively correlated with PIM-2, a score that predicts the risk of death for pediatric patients admitted to intensive care. Given biomarker changes that significantly correlate with illness severity, these biomarkers might be useful for sepsis prognosis.

Biomarkers correlating with PICU length of stay and overall hospital length of stay were also identified. Elastase 2, NGAL, and MIG positively correlated with PICU length of stay, whereas hyaluronan and MIG positively correlated with hospital length of stay. Elastase 2 and NGAL are released by activated neutrophils, MIG is a T-cell lymphocyte chemokine, and hyaluronan is cleaved from the microvascular endothelial glycocalyx. These biomarkers may represent a severe host-pathogen response as well as a prolonged recovery process.

Importantly, IL-3 was noted to be increased in pediatric patients who had a viral agent identified rather than a bacterial infection. IL-3 is produced by activated T-cells and increases innate antiviral immunity by promoting the recruitment of circulating plasma dendritic cells [43]. IL-3 may help distinguish which sepsis patients require antibiotic versus antiviral coverage and aid antimicrobial stewardship.

The importance of this study is highlighted by the number of blood biomarkers measured, together with advanced analytics. Indeed, recent pediatric sepsis biomarker studies focused on only a few biomarkers analyzed with conventional statistics [44–47]. The machine learning incorporated herein identified the biomarker importance for determining sepsis. We have also taken a novel approach to identify combinations of biomarkers that, when used together, yield potential utility in predicting sepsis severity.

The clinical relevance of this study is two-fold: first, identification of biomarker combinations may aid earlier recognition of sepsis in children; and second, the biomarker(s) that correlate with clinical variables and illness severity may aid prognostication. Quantitative laboratory and semi-quantitative point-of-care immunoassays could be constructed to measure multiple biomarkers identified here for early sepsis diagnosis, including multiplex, lateral flow, and chip assays. Future studies, using targeted proteomics and machine learning, should measure the plasma proteome in pediatric sepsis to identify novel biomarkers not yet considered, as has been done for adult sepsis [48].

Our study identified combinations of plasma biomarkers that act as significant markers of sepsis. Individually, the identified sepsis biomarkers share commonalities with other inflammatory conditions, including diabetic ketoacidosis [49, 50], trauma [51], and ischemic injury [52]. In contrast, biomarker combinations hold value for disease specificity and may allow for the distinction of sepsis from other inflammatory states, such as those listed above. Nonetheless, several limitations of our study are worthy of discussion. First, our study includes a limited sample size and was conducted at a single center. Despite this, the data yielded robust results even while using conservative statistical methods. Second, the pathogens were variable, with the majority suffering from bacterial illness as compared to viral pathogens [53]. Nonetheless, bacterial infections are amenable to antibiotic treatment, and early recognition and intervention are critical to improving outcomes [53]. Also, IL-3 seemed to distinguish bacterial from viral infections, thereby requiring further study. Despite these caveats, our exploratory data are important for future hypothesis-driven studies in larger populations.

## Conclusion

Our data have shown that elevated levels of certain biomarkers (specifically the combinations of IL-8 & IL-6, MCP-1 & HSP70, MCP-1 & IL-6, HSP70 & hyaluronan, and hyaluronan & IL-6) are potential markers of pediatric sepsis, and that elevations in certain biomarkers were correlated with clinical variables and illness severity measurements. These biomarkers are known to play a role in the mechanism of sepsis development [34–38]. As such, the combinations of these biomarkers that have been identified as significant by this study should be further investigated to identify their utility in specifically indicating the development of sepsis in pediatric patients versus other conditions causing inflammation. Furthermore, these biomarkers may also serve as a guide in clinical decision-making, and their early detection may eventually lead to improved outcomes in pediatric patients with sepsis.

### Electronic supplementary material

Below is the link to the electronic supplementary material.

Supplementary material 1

## Data Availability

The datasets generated and/or analysed during the current study are available from the corresponding author on reasonable request.
