# Supplementary material for: Pediatric sepsis inflammatory blood biomarkers that correlate with clinical variables and severity of illness scores
Source: J Inflamm (Lond). 2024 Mar 7;21:7. doi: 10.1186/s12950-024-00379-w (PMC10921642; doi:10.1186/s12950-024-00379-w)
Supplement: Supplementary file 1 — Supplementary material 1 [file 12950_2024_379_MOESM1_ESM.docx]

**Supplementary Table 1. Statistically Non-Significant Plasma Protein Measurements.**

| **Biomarker** | **Healthy Controls (n=20)** | **PICU Day 1 Sepsis (n=20)** | **PICU Day 2 Sepsis (n=13)** | **P-value** |
| --- | --- | --- | --- | --- |
| **IP-10** | 108.31 (60.05, 163.97) | 373.02 (145.14, 4321.94) | 263.19 (81.05, 3557.59) | 0.024 |
| **GM-CSF** | 0 (0, 0) | 2.86 (0, 34.87) | 12.42 (0, 74.17) | 0.029 |
| **Lactoferrin** | 433.01 (289.20, 543.97) | 239.23 (118.83, 468.18) | 269.90 (168.92, 421.27) | 0.028 |
| **IFNγ** | 4.04 (0.31, 7.91) | 14.75 (3.27, 31.71) | 8.08 (3.59, 20.80) | 0.047 |
| **IL-15** | 9.16 (3.63, 32.15) | 17.83 (10.88, 39.53) | 18.86 (10.07, 53.60) | 0.104 |
| **IL-12(p40)** | 90.93 (49.55, 138.52) | 53.53 (33.25, 132.96) | 52.40 (28.35, 96.70) | 0.147 |
| **Syndecan 1** | 118.60 (58.24, 353.12) | 316.52 (138.28, 534.29) | 264.75 (125.72, 761.12) | 0.157 |
| **Granzyme B** | 2.99 (2.42, 8.16) | 10.21 (2.90, 14.60) | 3.60 (2.33, 15.09) | 0.168 |
| **Thrombospondin-1** | 1008.78 (587.73, 1219.68) | 455.66 (209.23, 1026.87) | 540.89 (145.58, 1068.40) | 0.185 |
| **VEGFA** | 15.72 (4.97, 53.55) | 26.29 (16.54, 65.96) | 36.25 (15.65, 106.76) | 0.216 |
| **IL-7** | 0 (0, 0) | 0 (0, 0.48) | 0 (0, 0.60) | 0.224 |
| **IL-22** | 0 (0, 35.91) | 43.98 (0, 114.46) | 38.58 (0, 117.19) | 0.238 |
| **Heparin sulfate** | 25.16 (5.76, 61.49) | 7.15 (4.40, 33.85) | 5.96 (1.93, 59.42) | 0.239 |
| **MMP12** | 517.60 (369.87, 599.81) | 976.94 (283.05, 2207.68) | 547.32 (272.16, 788.15) | 0.321 |
| **IL-17E/IL-25** | 557.57 (185.72, 1174.19) | 685.27 (377.55, 2281.92) | 969.30 (303.59, 2563.56) | 0.358 |
| **TNFβ** | 0 (0, 8.84) | 2.78 (0, 17.87) | 0 (0, 14.87) | 0.414 |
| **IL-3** | 0 (0, 0.55) | 0 (0, 0) | 0 (0, 0.65) | 0.428 |
| **PDGF-AA** | 87.94 (56.16, 180.66) | 148.53 (53.40, 303.39) | 152.65 (43.14, 251.89) | 0.442 |
| **Eotaxin** | 57.57 (44.73, 67.04) | 62.14 (49.26, 105.46) | 59.01 (43.56, 71.83) | 0.477 |
| **PDGF-AB/BB** | 1264.83 (445.21, 1875.60) | 652.24 (0, 1933.33) | 656.25 (0, 1628.67) | 0.489 |
| **IL-17F** | 29.03 (11.10, 140.17) | 15.41 (4.34, 90.69) | 23.86 (2.42, 122.38) | 0.622 |
| **IL-2** | 0 (0, 0.94) | 0.04 (0, 1.87) | 0.20 (0, 1.97) | 0.625 |
| **IL-17A** | 8.80 (1.52, 20.91) | 4.30 (2.24, 10.50) | 3.40 (1.13, 11.61) | 0.650 |
| **IL-13** | 1.03 (0, 5.76) | 0.33 (0, 3.17) | 0 (0, 7.76) | 0.668 |
| **Chondroitin sulfate** | 9.29 (6.08, 15.00) | 7.86 (0.44, 20.99) | 10.32 (3.86, 27.88) | 0.701 |
| **IL-12(p70)** | 2.88 (0, 9.54) | 2.74 (0.72, 6.84) | 1.91 (0, 7.78) | 0.746 |
| **IFNα2** | 17.21 (2.11, 32.30) | 11.67 (5.14, 20.99) | 16.62 (4.92, 24.84) | 0.755 |
| **MIP-1α** | 20.92 (0, 75.25) | 34.31 (6.40, 73.36) | 28.28 (0, 87.41) | 0.763 |
| **IL-1α** | 6.01 (1.77, 14.87) | 4.03 (1.54, 10.05) | 5.37 (2.10, 12.54) | 0.779 |
| **IL-4** | 0.08 (0, 4.06) | 0.45 (0, 2.68) | 0.41 (0, 6.46) | 0.865 |
| **IL-5** | 6.52 (3.01, 12.98) | 6.39 (2.66, 13.44) | 7.09 (2.51, 17.37) | 0.920 |
| **IL-1β** | 13.94 (3.68, 24.38) | 10.45 (6.77, 20.70) | 9.26 (4.80, 24.13) | 0.949 |
| **MMP13** | 692.19 (247.51, 1346.71) | 835.61 (228.91, 2336.98) | 812.89 (477.96, 1098.34) | 0.965 |
| **EGF** | 5.20 (0, 20.88) | 7.61 (0, 15.55) | 8.97 (0, 25.27) | 0.989 |

Data presented in pg/mL as medians (IQRs). P<0.01 was considered significant.
